# Stand-Alone GNSS Sensors as Velocity Seismometers: Real-Time Monitoring and Earthquake Detection

**DOI:** 10.3390/s18113712

**Published:** 2018-10-31

**Authors:** Roland Hohensinn, Alain Geiger

**Affiliations:** ETH Zurich, Institute of Geodesy and Photogrammetry, 8093 Zurich, Switzerland; alain.geiger@geod.baug.ethz.ch

**Keywords:** high-precision GNSS, instantaneous GNSS velocity, significance testing, GNSS seismology, geohazard monitoring, seismic monitoring, earthquake early warning

## Abstract

By means of the time derivatives of Global Navigation Satellite System (GNSS) carrier-phase measurements, the instantaneous velocity of a stand-alone, single GNSS receiver can be estimated with a high precision of a few mm/s; it is feasible to even obtain the level of tenths of mm/s. Therefore, only data from the satellite navigation message are needed, thus discarding any data from a reference network. Combining this method with an efficient movement-detection algorithm opens some interesting applications for geohazard monitoring; an example is the detection of strong earthquakes. This capability is demonstrated for a case study of the 6.5 Mw earthquake of October 30, 2016, near the city of Norcia in Italy; in that region, there are densely deployed GNSS stations. It is shown that GNSS sensors can detect seismic compressional (P) waves, which are the first to arrive at a measurement station. These findings are substantiated by a comparison with data of strong-motion (SM) seismometers. Furthermore, it is shown that the GNSS-only hypocenter localization comes close (less than a kilometer) to the solutions provided by official seismic services. Finally, we conclude that this method can provide important contributions to a real-time geohazard early-warning system.

## 1. Introduction

High-precision estimates of the instantaneous velocity of a GNSS sensor (‘GNSS sensor’, ‘GNSS receiver’, and ‘GNSS station’ are used as synonyms) can be obtained based on time derivatives of GNSS phase measurements. This technique of GNSS velocity determination has been discussed for almost two decades in the geodetic literature, with a focus on applications in navigation [1,2,3,4,5,6]. An advantage is its use in “stand-alone mode”, where no auxiliary data from a reference network are needed, and it can be operated with single- as well as with multifrequency receivers. The authors of the publications listed above state levels of accuracy of some mm/s. For most applications, the sampling rate is set to 1 Hz or higher. However, the higher the sampling rate is set, the higher the noise level will be [7]. It was also recently shown that, with multi-GNSS measurements, accuracies at the level of few tenths of mm/s are possible for the most precise component of the three-dimensional velocity vectors [7,8]. A statistical framework was set up to test the estimated velocity epochwise for significance, based on the signal-to-noise (SNR) ratio of velocity w.r.t. to covariance. Whenever this quantity exceeds a certain threshold, a movement is detected. By introducing statistical criteria that extend over several epochs, decision making becomes more reliable. However, this algorithm enables a GNSS receiver to be used as a velocity seismometer for seismic monitoring in real time, and hazardous movements can be rapidly detected (with a delay of few epochs). For the dataset under investigation it is also shown that, depending on the station’s epicentral distance, the arrival of two different seismic phases can be resolved. The arrival of P-waves is validated by a comparison with a dataset of strong-motion accelerometer records, since these have much higher sensitivity to small movements compared to GNSS. Finally, it is demonstrated that the arrival times determined by GNSS allow for an estimation of the hypocenter coordinates. To this end, a simple inversion model is set up, with the arrival times as an input, and the hypocenter coordinates (in WGS84) and origin time as unknowns.

Applications of real-time GNSS seismology mainly focus on the computation of dynamic displacements from kinematic GNSS applications, like Real Time Kinematic (RTK) or Precise Point Positioning (PPP) [9], as demonstrated by Ohta et al. [10] or Xu et al. [11]. Typically, sampling rates ≥1 Hz are of interest. The obtained precision is usually at the level of few centimeters (RTK-GNSS) down to the sub-cm level, as stated for an application of PPP [11]. It was demonstrated that the Global Positioning System (GPS) is capable of resolving seismic surface waves and shear (S) waves [12,13]. Compared to seismometers, the advantage of GNSS measurements is that they do not suffer from saturation effects [14]; thus, they are well suited for magnitude estimation of strong earthquakes. Furthermore, the coseismic displacements from GNSS do not have to be obtained from the integration of acceleration or velocity measurements, as for most seismometer records. When using RTK or PPP for seismology, the correction data from reference stations may be corrupted by the ground shaking. For RTK, this can be compensated by forming long baselines. However, the accuracy of the estimates may then be degraded. For PPP, the real-time orbit and clock products could also be affected, since base stations located near the earthquake might also be displaced. Furthermore, PPP still needs convergence times of about 15–30 min or more in order to reach centimeter-level accuracy. To partially overcome problems of convergence, methods have been developed that focus on the estimation of relative displacements (“Delta Positions”) with single GNSS receivers. An algorithm called G-MoDe (GPS Movement Detection) computes relative displacements based on short-term predictions of GPS L1 carrier-phase measurements [15,16]. Displacements down to few mm could be resolved epochwise. Colosimo et al. [17] presented the “variometric” approach: The coseismic slip can be computed by detrending integrated instantaneous GNSS velocities. Another method, which derives relative displacements from a modified PPP algorithm (with the coordinates of a fixed reference epoch) was introduced by Li et al. [18]. It was shown that these two methods are mathematically equal, and the results were compared with a converged PPP solution in [19]. For coseismic displacements of the 2011 Tohoku earthquake, it was shown exemplarily that the differences with regard to the integrated relative positions were at only the level of few cm.

Over the last decade, real-time GNSS applications have become an important field of research for earthquake early warning (EEW). Crowell et al. [20] developed an EEW system that estimates hypocenter, magnitude, and source model parameters. They used GPS total displacement waveforms with a detection algorithm relying on a threshold criterion. The detection algorithm of Ohta et al. [10] is based on a Short-/Long-Term Average (STA/LTA) criterion, which is applied to GPS time series in order to rapidly detect static displacements; STA/LTA algorithms are commonly used for detecting earthquakes in seismometer records. Crowell et al. [20] also estimated the coordinates of the hypocenter for the 2003 8.3 Mw Tokachi-Oki earthquake, and obtained results taht were several tens of kilometers away from the official seismic solutions. Currently, several operational GNSS-based EEW are under development, e.g., G-FAST for Chile [21] or REGARD in Japan [22]. They typically aim at fast earthquake detection, magnitude estimation, and determination of the coseismic slip, as well as the determination of finite fault models and moment tensors. An optimal way is to use both GNSS and seismometer data for EEW: According to Allen and Ziv [23], a proper way is to derive magnitude from the GNSS, and to perform detection and localization with seismometer data. Another example is sensor fusion: Goldberg and Bock [24] combined GNSS and strong-motion accelerometer data in a Kalman filter, and estimated hypocenter, magnitude, and coseismic offset. Finally, Grapenthin et al. [25] obtained instantaneous velocity estimates from single-frequency GNSS data for the Mw 7.1 Iskinsin Alaska earthquake. Accuracies of some mm/s were obtained, and S-waves could be resolved.

This work aims at filling existing gaps for GNSS earthquake early-warning systems: Instantaneous GNSS velocity estimation can very rapidly provide movement information (no convergence time, low infrastructural costs) utilizing statistical testing. This investigation also demonstrates the capability of resolving different seismic waves, especially the first arrivals (P-phase). Based on this, the potential of GNSS-only hypocenter estimation is also highlighted. The paper is organized as follows: Section 2 introduces the principles of instantaneous GNSS velocity estimation, and the movement detection algorithm is reviewed. Furthermore, the framework for GNSS-only hypocenter localization is introduced. Section 3 shows the results of the detection for the example of the Mw 6.5 event in central Italy on October 30, 2016. The results are compared with data from a strong-motion (SM) seismometer network. Finally, the hypocenter and the origin time are estimated. The main findings are summarized and discussed in Section 4.

## 2. Methodology

### 2.1. GNSS Instantaneous Velocity

A very precise GNSS velocity observation equation can be obtained by the time differentiation of the carrier-phase observation equation when converted to units of meters [3,4,8]. The resulting velocity is in receiver-to-satellite line of sight (LOS), and primarily consists of LOS receiver and satellite velocity, receiver and satellite clock drifts, ionospheric and tropospheric rates, relativistic effects, as well as multipath and observation noise (the unknown phase ambiguities are eliminated by time differentiation). Prominent effects, like satellite velocity, satellite clock drift, and atmospheric and relativistic effects can mostly be accounted for by models with information from the satellite broadcast message. The remaining effects are summarized in a reduced velocity observation equation [7,8]:

(1) $$\begin{array}{cc} v_{r,RED}^{i}\left(t\right) & ={\left(v_{r}\left(t\right)\right)}^{T}a_{r}^{i}\left(t\right)-c{\dot{\delta t}}_{r}\left(t\right)+{\dot{\epsilon }}_{i}\left(t\right)\\ & =v_{r,x}\left(t\right)a_{r,x}^{i}\left(t\right)+v_{r,y}\left(t\right)a_{r,y}^{i}\left(t\right)+v_{r,z}\left(t\right)a_{r,z}^{i}\left(t\right)-c{\dot{\delta t}}_{r}\left(t\right)+{\dot{\epsilon }}_{i}\left(t\right) \end{array}$$

$v_{r,RED}^{i}\left(t\right)$ is the reduced velocity observation (m/s) from receiver *r* to satellite *i* at GNSS system time *t*, $v_{r}\left(t\right)$ is the receiver velocity vector (xyz-components, WGS84), $a_{r}^{i}\left(t\right)$ is the receiver-satellite LOS unit vector, *c* is the speed of light, $\delta {\dot{t}}_{r}\left(t\right)$ is the receiver clock drift and ${\dot{\epsilon }}_{i}\left(t\right)$ is the observation error, which consists of remaining biases and the time derivative of the measurement noise. A system of linear observation equations l=Ax can then be set up per epoch (l is the vector of observations), and inverted by means of weighted least-squares; the columns of observation matrix A (design matrix) are made up by LOS unit vectors, as well as an entry for the clock drift parameter. This system can be solved either in batch mode or recursively (e.g., Bar-Shalom et al. [26], Leick et al. [27]). There are four parameters estimated for each epoch: the three components of receiver velocity vector $v_{r}\left(t\right)$ (in WGS84), as well as the receiver clock drift. This results in parameter vector $\hat{x}$ and covariance matrix $Q_{\hat{x}}$ with dimension 4×1 and 4×4, respectively.

### 2.2. Detection of First Arrivals

Epochwise movement detection is based on the statistical significance test of the signal-to-noise ratio of the vector of estimated receiver velocity vector $\hat{v}$ and its covariance matrix $Q_{\hat{v}}$. The test quantity is the quadratic form [8]:

(2) $$T={\hat{v}}^{T}Q_{\hat{v}}^{-1}\hat{v}$$

with the null hypothesis

(3) $$H_{0}:\;T\leq \chi_{\alpha }^{2}\left(3,0\right)$$

which is expected to be Chi-square-distributed with a degree of freedom of three and a noncentrality parameter of zero for a chosen level of significance α. This means that, whenever the test level is exceeded, a movement is detected. The number of false alarms depends on the chosen level of significance and the strength of unmodeled signals. To enhance a reliable decision, a criterion covers multiple epochs. A possible way is to extend test quantity *T* by including several epochs, which then becomes $\sum^{k}{\hat{v_{k}}}^{T}Q_{\hat{v_{k}}}^{-1}\hat{v_{k}}$, with 3×k degrees of freedom. Another possibility is to form a test quantity with a cumulative relative frequency criterion. Based on the epochwise test of Equation (2), a boolean is specified:

(4) $$b_{n}^{mov}=\left\{\begin{array}{cc} 1\;\;\ldots \;\; & \mathrm{if}\;T>\chi_{\alpha }^{2}\left(3,0\right)\;\\ 0\;\;\ldots \;\; & \mathrm{if}\;T\leq \chi_{\alpha }^{2}\left(3,0\right)\; \end{array}\right.$$

*n* is the index of the current epoch, and *T* the test result of the instantaneous movement detection test of Equation (2). The cumulative relative frequency over a sliding window of length *N* can then be computed by

(5) $$P_{n}=\frac{1}{N}\sum_{k=0}^{N-1}b_{n-k}^{mov}$$

Based on a threshold in the interval $0\leq P_{n}\leq 1$, a decision is made:

(6) $$Movement=\left\{\begin{array}{cc} \mathrm{Yes}\;\;\ldots \;\; & \mathrm{if}\;P_{n}\geq \;\mathrm{threshold}\\ \mathrm{No}\;\;\ldots \;\; & \mathrm{if}\;P_{n}<\;\mathrm{threshold} \end{array}\right.$$

When the test is positive, the first arrivals can be assigned to a time stamp $t_{n}$ for each station. Within the window of length *N*, this time stamp is then set at the first epoch at which the movement detection test is positive. For the following case study, level of significance α was set to 0.5%, and, for the cumulative relative frequency criterion, 7 out of 8 movements had to be detected in order to set the movement flag ($P_{n}\geq 7/8$).

### 2.3. GNSS-Only Localization

The hypocenter estimation is based on a simple 3D intersection model, with origin time as an additional parameter, and detected arrival times $t_{j}$ (for each station *j*) as observations:

(7) $$t_{j}=\frac{1}{v_{j}^{s}}\sqrt{{\left(x_{j}-x_{0}\right)}^{2}+{\left(y_{j}-y_{0}\right)}^{2}+{\left(z_{j}-z_{0}\right)}^{2}}+t_{0}+\sigma_{j}$$

$x_{j}$, $y_{j}$, and $z_{j}$ are the GNSS station coordinates, and $x_{0}$, $y_{0}$, and $z_{0}$ the hypocenter coordinates. Origin time $t_{0}$ and the hypocenter coordinates are the parameters (unknowns) to be estimated. $\sigma_{j}$ is the observation uncertainty of the arrival time, and $v_{j}^{s}$ represents seismic velocity. It is noted that, by an exchange of $v_{j}^{s}$ with speed of light *c*, this equation corresponds to the most simple form of the GNSS measurement equation (e.g., Hofmann-Wellenhof et al. [28]). It is also solved by means of weighted least-squares adjustment. The stochastics of the accuracy of arrival times $t_{j}$ is modelled by

(8) $$\sigma_{j}=\sigma_{0}\;+\;\sigma_{0}\;\left(\frac{d_{j}^{2}}{d_{ref}^{2}}\right)$$

where $\sigma_{0}$ is the variance of the unit weight, $d_{ref}$ is a hypocentral reference distance, and $d_{j}$ is the hypocentral distance to station *j*. Equation (8) is based on the weighting scheme that is applied by the Japan Meteorological Agency [29].

## 3. Case Study

### 3.1. Data

The dataset under investigation is the 6.5 Mw earthquake of October 30, 2016 that occurred near the city of Norcia in the Apennines (central Italy). There, the Italian Instituto Nazionale Geofisica e Vulcanologia (INGV) operates a dense network of GNSS stations, from which raw measurement data are freely available, provided by Rete Integrata Nazionale GPS (RING) [30]. As an example, these data were also processed by Avallone et al. [31] in order to obtain high-rate coseismic displacements from GPS for the 6.0 Mw event of August 24, 2016. The sampling rates of the stations range from 1 to 20 Hz. For this work, the GPS data of 42 stations have been processed. In order to get a good trade-off between GNSS velocity observation noise and the resolution of the movement dynamics, data with sampling rates higher than 2 Hz have been downsampled to 2 Hz. Finally, the datasets consist of 14 stations sampled at 1 Hz, and 28 stations sampled at 2 Hz, located at epicentral distances ranging from about 10 to 170 km. For comparison, and as a ground reference, the most recent values for the earthquake hypocenter as well as for the origin time were taken from the Centro Nazionale Terremoti (CNT) of INGV (http://cnt.rm.ingv.it/en/event/8863681, last accessed September 9, 2018). They are listed in Table 1. The precision of the earthquake hypocenter is specified with values of around 100–200 m for the axis of the confidence ellipsoid.

### 3.2. GNSS Seismograms, First Arrivals, and Seismic Velocities

**Figure 1:**
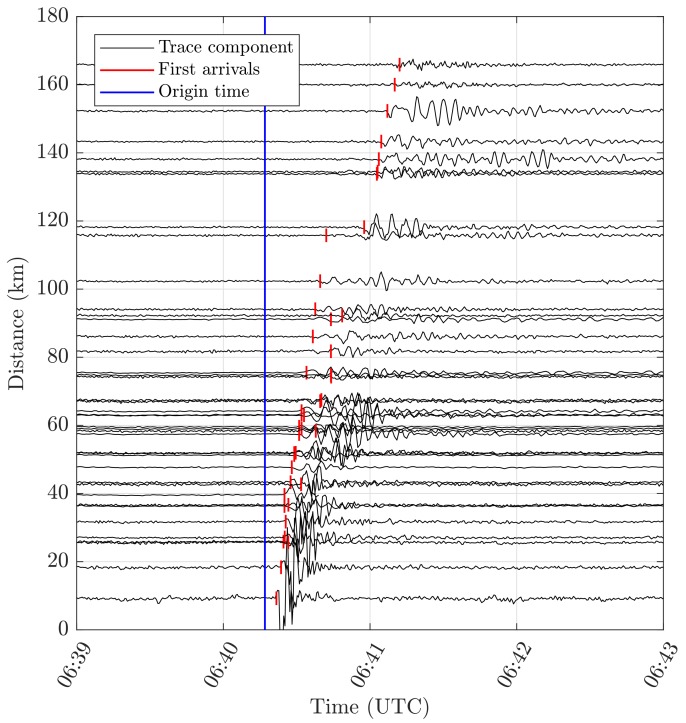
Seismograms of the North velocity component of the Global Navigation Satellite System (GNSS) stations with regard to the station’s epicentral distance, as well as the detected first arrivals from GNSS and the reference origin time. The maximum magnitudes of station velocity lay between 0.5 and 0.01 m/s for the closest and the farthest station, respectively.

**Figure 2:**
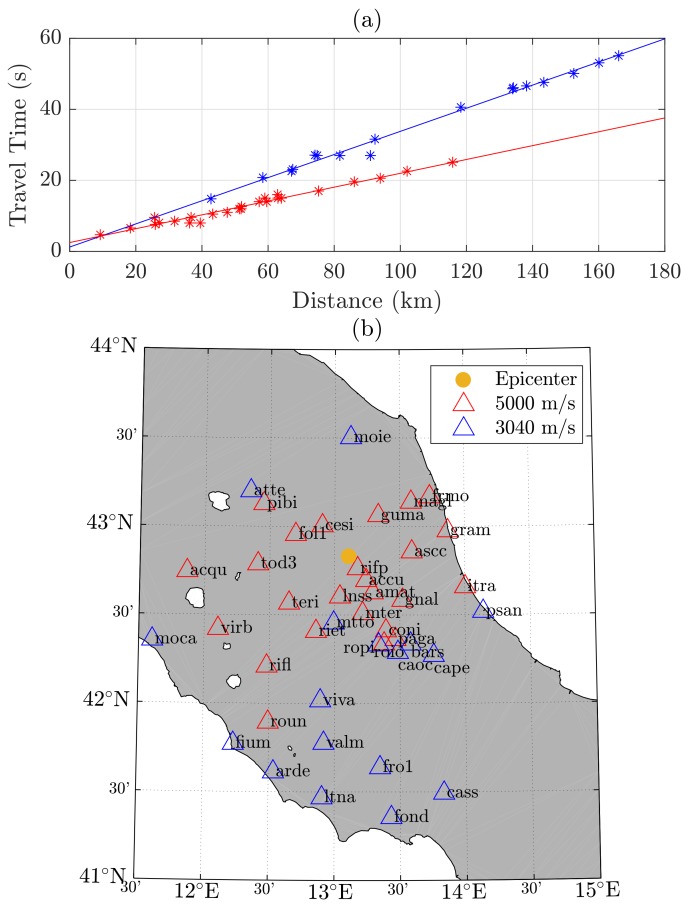
Plot (**a**): Seismic travel times from GNSS (w.r.t. reference origin time) with regard to epicentral station distances, and regression lines for the two seismic phases that were identified. Plot (**b**): GNSS stations with the seismic phases of plot (**a**) assigned. The observed radiation pattern agrees well with the reported fault characteristics.

The components of the instantaneous GNSS receiver velocity, as well as the receiver clock drift, were estimated epochwise in a batch solution based on Equation (1). The covariance matrix was computed by $Q_{\hat{x}}={\left(A^{T}Q_{l}^{-1}A\right)}^{-1}$, where $Q_{l}$ is the variance matrix of the reduced velocity observations. The variance of the observations was determined based on the Root Mean Square Error (RMSE) of the velocity residuals of a static phase right before the event; the observations were assumed to be uncorrelated. From $\hat{x}$ and $Q_{\hat{x}}$, the partition of the velocity components was then extracted and the movement-detection test was carried out. The velocity components were then transformed to the topocenter (East, North, Up frame) for each station. Figure 1 shows a 4 min window of the seismic traces of the stations’ North velocity component, together with the detected first arrivals and the reference origin time. Seismic waves can clearly be identified in all 42 stations. The maximum magnitudes of station velocity lay between 0.5 and 0.01 m/s for the closest and farthest station, respectively. At the detected first arrivals, the GNSS minimum detectable velocities lay between 1.8 and 4 mm/s. They were computed for a test power of 50% and a level of significance of 0.1% (in accordance with the level of 0.5% for the Chi-square test) [7]. Figure 2a depicts the seismic travel times (“GNSS arrival times minus reference origin time”) with regard to the station’s epicentral station distance. Two seismic phases could be identified, and individual stations were assigned to one of them. A regression line was then fitted to each group. The corresponding velocities were obtained at around 3040 and 5000 m/s. These velocities are associated with the arrivals of S- and P-waves, respectively. A comparison with 1D crustal velocity models of the region reveals similar results: as an example, an average of a 1D-velocity model of Herrmann et al. [32] for this region (weighted by layer thickness) gave values of 2950 and 5150 m/s, respectively. Figure 2b shows a map of the GNSS stations, with each station colored by the corresponding seismic phase. The observed radiation pattern agrees well with the reported fault characteristics [33]. This is typically a normal faulting mechanism for the region of the central Apennines, with strike angles of around 150–160 degrees, and dip angles of around 45 degrees. Fault plane solutions for historical earthquakes in this region can also be found in Michele et al. [34].

### 3.3. Validation of GNSS First Arrivals

**Figure 3:**
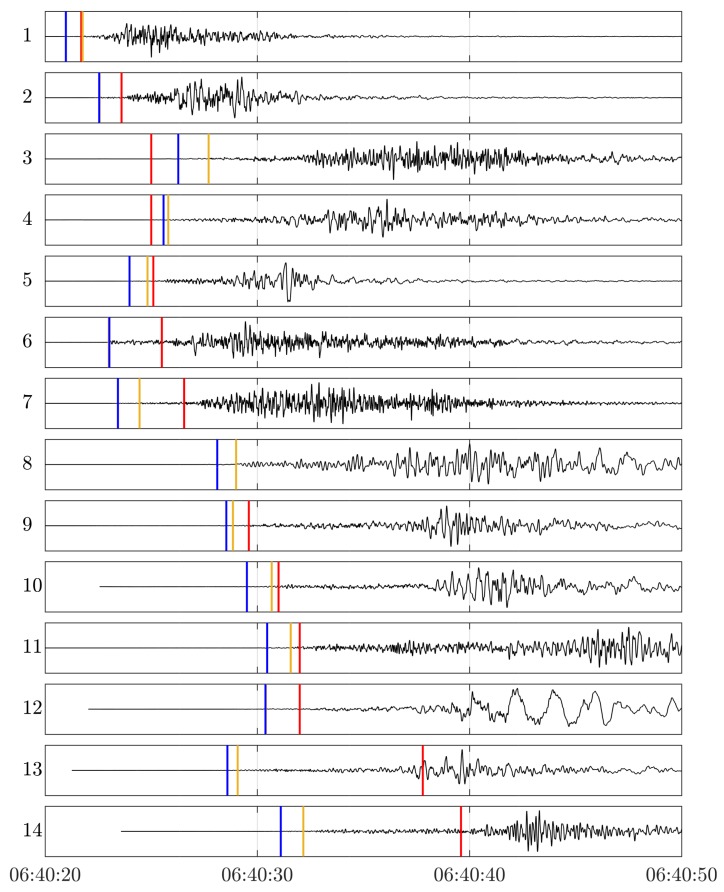
Seismograms of the radial acceleration component for the strong-motion (SM) stations, which are colocated with GNSS stations at distances smaller than 5 km. These sites are marked with ID 1–14. The vertical bars indicate the detected first arrivals: Red for GNSS, light brown for the SM radial component (automatic picking), and blue for the SM radial component (manual picking).

For obtaining a reference for the detected first arrivals from GNSS, the engineering strong-motion (SM) seismometer dataset provided by the Observatories and Research Facilities for European Seismology (ORFEUS) [35,36] was used. For the event under investigation, there were data for 100 stations, which were located at distances of around 5 to 100 km from the epicenter. For this dataset, 14 quasi colocated stations could be identified based on the criterion that the interstation distance of the GNSS and seismometer stations was smaller than 5 km. For these sites, the seismograms of the SM acceleration components (East, North, and Up) were then projected into a source-centered radial system (Radial, Transversal, Vertical). The first arrivals of the P-waves were then both manually and automatically picked from the radial acceleration component. For automatic picking, the P-phase picker of Kalkan [37] was utilized, which was developed at the United States Geological Survey (USGS). The seismograms of the SM radial acceleration components for the 14 colocated stations can be found in Figure 3, together with the first arrivals. The stations are sorted with regard to GNSS arrival times (red lines).

**Figure 4:**
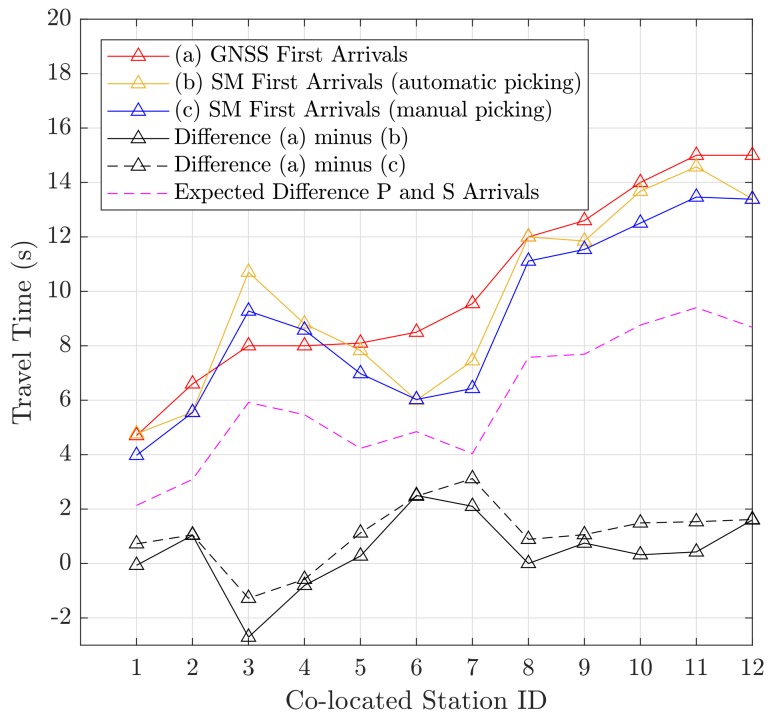
Seismic travel times (w.r.t. reference origin time), and the differences in travel times of GNSS versus the seismometer arrivals. The purple line shows the expected time difference of P and S arrivals. A small delay of the GNSS arrivals can be seen. Almost exclusively, first arrivals could be assigned to P-phase arrivals.

Figure 3 reveals that almost all detected first arrivals could be assigned to the P-phase. S-waves typically travel at a velocity of around 2/3 of the P-wave, and are thus expected to arrive right before the surface waves (for shallow earthquakes). An exception are the GNSS arrivals at Stations 13 and 14, which can be associated with the slower phase detected with GNSS (see Figure 2a). These are close to S-wave arrivals. The differences between the first arrivals of Stations 1–12 are shown in Figure 4.

**Figure 5:**
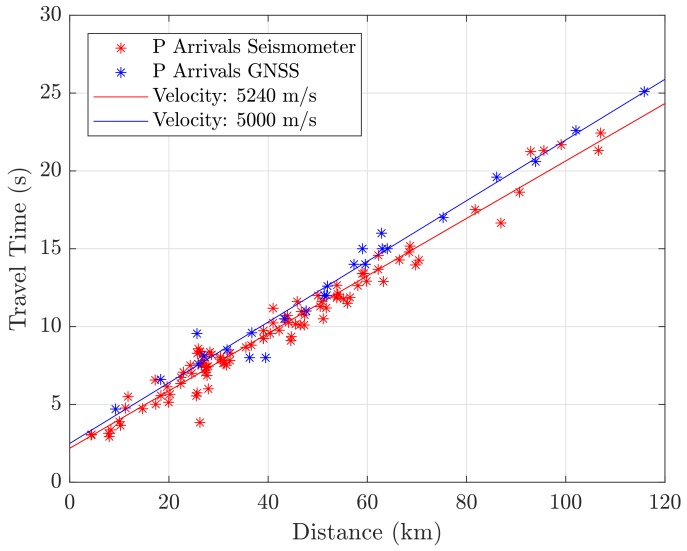
Travel times and velocities of P-wave arrivals obtained for GNSS, and for all seismometer stations, respectively. There is a tendency for the GNSS arrivals to be detected with a small delay compared to the first arrivals of the seismometers.

It can be noticed that, in general, GNSS detects first arrivals with a small delay compared to the SM arrivals. This is likely to be a result of the lower sensitivity of GNSS sensors. The RMSE/median of the differences is 1.4/0.4 s for the automatically picked SM arrivals and 1.6/1.1 s for the manually picked SM arrivals. The delay is smaller than two seconds, except for colocated Sites 6 and 7. It should be noted that biases in arrival times between the GNSS and SM picks can also result from eccentric stations: an eccentricity of 5 km can cause an offset of around 1 second between the arrival times of the P-waves. This especially applies to Stations 3 and 6, where the epicentral station difference was about −4 and 4 km, respectively. However, for all stations (except Stations 6 and 7), the differences are at least three times smaller than the expected time difference between the P-wave and the S-wave arrivals, and first arrivals can thus be assigned to the P-phase. Figure 5 shows the travel times for the GNSS first arrivals (P-waves), and the same for the automatically detected first arrivals of all seismometer stations. It can be seen that there is a small misalignment between the regression lines, which also results in different velocities. This means that, at larger distances, GNSS obviously tends to detect P-waves later, which can also be addressed to sensitivity issues, and as a consequence of wave-propagation effects like scattering and attenuation. This also emphasizes the findings for the colocated stations.

### 3.4. Localization

**Figure 6:**
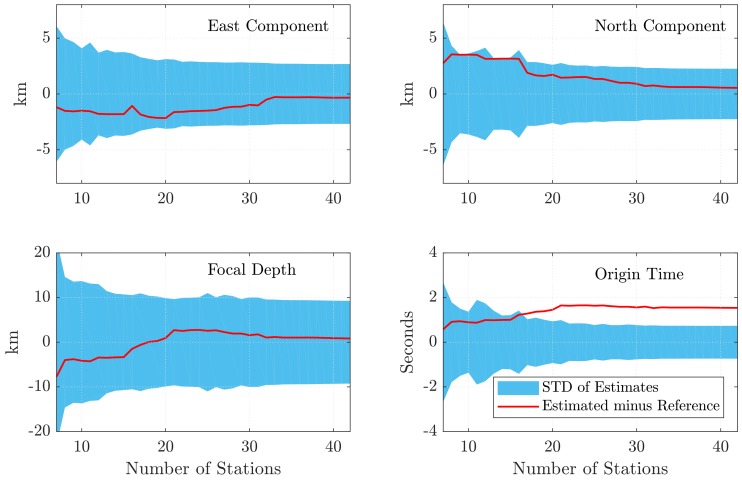
Differences of the estimated earthquake hypocenter from the GNSS arrivals with regard to to the reference hypocenter coordinates (for the East, North, and Depth components), as well as the differences of the estimated origin time with regard to the reference. Overall, the co-ordinates obtained for the hypocenter agree within 1–2 km of the reference solution, and better than one kilometer for the final estimates including all 42 stations.

For the initial solution of Equation (7), the detected arrival times of the first seven stations were taken. The approximate values of the parameters for the first adjustment were more than 15 km off the reference values for the co-ordinates, and around 4 s off for the origin time, respectively. Then, sequentially with each additional detected arrival, another solution was computed until all 42 stations were included. Seismic velocities $v_{j}^{s}$ were assigned according to the results of the regression of Figure 2a. In Equation (8), we set $\sigma_{0}$ to 1 second, which is approximately the RMSE of the residuals for the result of the GNSS velocity regression (see Figure 2); $d_{ref}$ was set to 50 km. For the final set of estimates, this resulted in a sigma of about 1 second for the first station, up to two seconds for all stations up to 50 km, around 5 s up to 100 km, and the stations farthest away then do have a σ of around 10 s. The results of the estimation procedure are presented in Figure 6: The estimated hypocenter coordinates (xyz, WGS84) were transformed to geodetic latitude, longitude, and ellipsoidal height (focal depth; geoid undulation was neglected). Then, the differences of geodetic latitude and longitude with regard to the reference solution were computed and transformed to kilometer units (multiplication by 111 km, and 111 km times the cosine of the latitude, respectively). In addition, the standard deviation band of the estimates is shown. Figure 6 shows the 3D localization converges towards the reference values with increasing number of stations. The mean departures are about −1.1, 1.7, and 0.7 km (East, North, Depth), and better than 1 km for the final set of estimates with 42 stations. The estimate of the origin time shows a mean offset of around 1.4 s (1.5 s for the last estimate). This is probably caused by the small delay in the detection of the P-arrivals with GNSS, which is then absorbed by the estimate of the origin time.

## 4. Conclusions

It was demonstrated that estimates of the GNSS instantaneous receiver velocity can resolve seismic signals down to amplitudes of a few millimeters per second. With a dense network of GNSS stations, we were able to detect arrivals of the P-phase of seismic waves of a strong earthquake in central Italy, with each station processed autonomously. Based on the detected GNSS first arrivals, an estimation of the earthquake hypocenter was carried out. For this case, it was shown that, even with a simple model, the estimated hypocenter can converge to the reference solution from seismology. There are open issues: The phases of the detected waves have to be reliably identified, so that a priori coordinates with sufficient accuracy can be provided, and that the accuracy stochastics of the arrival times can be set up properly and in an automated manner. Robust estimation methods (e.g., M or L estimators) might be well suited to address these issues. Likewise, as for GNSS positioning, the height (depth) component is most sensitive to modeling errors. A way to address this problem would be to use a priori constraints, e.g., on the seismic velocities or on the depth, since most strong earthquakes in this region occur at depths of up to around 15 km [38]. It is important to mention that the proposed algorithm still has practical limitations; their main aspects are the spatial density of the GNSS stations, the earthquake magnitude, and the sensitivity of the GNSS: As a rule of thumb, at a 1 or 2 Hz sampling rate and GPS-only observations, the sensitivity of the receiver velocity estimates is at the level of few mm/s. The reliable detection of an earthquake requires about 5 to 10 consecutive epochs of significant estimates of ground velocity. The functionality of the method, as well as the full processing flow, including the detection of P-arrivals and localization, were successfully applied to a strong earthquake (>6 Mw), ideally monitored by a high spatial density of (several tens of) GNSS stations, at a spatial distribution of tens of kilometers. In poorer configurations, earthquakes of moderate size (>5 Mw) can still be detected, but phase identification and localization might then no longer be possible. Planned-applicability studies can further investigate peak ground-velocity (e.g., Akkar and Bommer [39]) detection in different GNSS network configurations. Local densely deployed GNSS stations in high-risk areas can be of double benefit: On the one hand, for earthquake detection and localization by the proposed algorithm, and, on the other hand, for revealing detailed information of the coseismic and postseismic slip obtained from GNSS coordinate series. Due to its real-time capability, the presented algorithm has the potential to provide independent and fast information for EEW. It could help enhance EEW systems based on seismometer networks. Ongoing work focuses on the GNSS minimum detectable velocities depending on earthquake magnitude and station distance, and on a characterization of the detected seismic phase. Results for multi-GNSS experiments give very promising results, since they enable detection of signals at the sub-mm/s level. It can be stated that this GNSS method can provide an autonomous solution for geohazard monitoring, and can thus augment an earthquake early-warning system.

## Figures and Tables

**Table 1:** Earthquake hypocenter coordinates and origin time (from Centro Nazionale Terremoti-Instituto Nazionale Geofisica e Vulcanologia (CNT-INGV)).

Date and Time (UTC)	Moment Magnitude	Latitude (deg)	Longitude (deg)	Depth (km)
2016/10/30 06:40:17	6.5	42.83	13.11	10
